# Clustering Algorithm in English Language Learning Pattern Matching under Big Data Framework

**DOI:** 10.1155/2022/1380046

**Published:** 2022-09-06

**Authors:** Liying Zheng

**Affiliations:** Inner Mongolia Vocational and Technical College of Communications, Chifeng 024005, Inner Mongolia, China

## Abstract

The Internet era has brought new challenges and opportunities for English learning and English teaching. At the same time, basic education is fully implementing quality education and respecting students' individual differences. The same teacher teaches the same content to the same class of students, but some students perform well, and some students perform poorly due to the influence of intellectual and nonintellectual factors. The uneven performance of students in the same class makes it very difficult for teachers to teach. In view of the current situation of university English teaching and the trend of respecting students' individual development in the new era, this study investigates the basic concept of English language learning pattern matching, its main features, and practical application in the process of university English teaching. The clustering algorithm based on the big data framework is proposed for English language learning pattern matching, which is fault-tolerant and can quickly acquire and process the big data information in English teaching. By analyzing the characteristics of the data mining method of students' English learning behavior, the method of clustering processing for students' English learning data mining and the processing method of students' English learning clustering data are explored. The method is highly adaptable and can be used for actual English language learning pattern matching, and actively explores the main path of English teaching change and innovation.

## 1. Introduction

**Figure 1:**
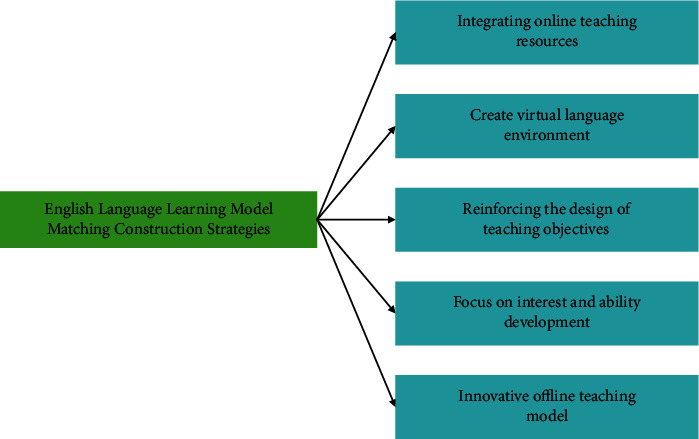
English language learning pattern matching schematic.

The application of new technologies, methods, and means in various industries in the background of the Internet era has promoted the rapid development of enterprises in China. The requirements of enterprises for talents in the process of development are constantly transforming, and some enterprises now have certain requirements for talents' English, making university English teaching a hot topic. How to change the teaching concept, enrich the teaching methods, and use modern new teaching methods to improve the classroom teaching effect according to the needs of enterprise talents is the problem that English teachers need to solve. The use of English language learning mode matching is decided according to students' characteristics because of their different levels of English. The diversity of students' enrollment methods leads to a large difference in students' English levels, and the use of English language learning mode matching has been proven to meet the development needs of modern English teaching [1–3]. In the process of English language learning mode matching centered on students' development, it is necessary not only to improve students' basic level of English, but also to improve the function of English education and professional services, which effectively improves the quality of classroom teaching and brings into play the function of English teaching and education of people. The schematic diagram of English language learning pattern matching is shown in Figure 1.

Problems of English teaching in the background of the Internet. Personalized teaching is missing. Nowadays, students are all post-00s, and teachers need to change their teaching philosophy and update their teaching methods in the process of teaching to meet the needs of students' personalized development and to play a role in promoting students' innovation ability. Teachers need to combine the characteristics of students to carry out teaching design, but some teachers do not have scientific teaching design, teaching links are not complete, and do not consider the needs of students' personalized development. College English is not only to improve students' basic English level but also needs to serve students' professional development according to their professional development [4–8]. The goal of teaching reform is to suit students' development, combine the requirements of students' professional job abilities, continuously explore teaching reform, update teaching concepts, and integrate new knowledge, methods, and means into teaching to provide the possibility of meeting students' personalized development. The huge amount of learning resources increases confusion. In the era of Internet+, there are many online platforms, but the quality of each platform is different, resulting in many online teaching resources, but students' independent learning ability is weak, resulting in students not knowing those resources to help students' English learning, and the huge amount of learning resources increases confusion.

Teachers need to guide students in the process of teaching to choose learning resources, let students go to the designated platform to choose the corresponding learning resources, upload the needed learning materials through flipped classroom, and so on. The teachers can let students fully prestudy, which is an effective guarantee to improve the quality of classroom teaching. Choosing teaching resources according to the guidance of teachers can improve the efficiency of students in choosing teaching resources and promote students to use network teaching resources to learn. Many non-national dual first-class teaching institutions, students have a weak English foundation, even in the local dual first-class professional teaching institutions, but individual institutions enrollment quality is also bad, the quality of students is also declining, the quality of the rest of the student population is even worse, the overall level of students in English is not high, it is difficult to adapt to continue learning mode, which must be based on student characteristics to take English language learning mode match, which is to ensure the English classroom an important factor to ensure the quality of teaching [9–14].

Internet era, many online platforms, and teachers guide students in online learning, but now few students can complete the learning independently, and need teachers scientific guidance, supervision, and counseling to complete the online teaching tasks and a plan to enhance students' self-learning ability. English is an important subject in the process of student development, now companies have certain requirements for students' English application skills, especially foreign-funded enterprises, to the international direction. The development of enterprises has certain requirements for the application of English knowledge of talents, and it is necessary to train students' online learning ability according to their characteristics, so as to lay the foundation for their learning and meet the development needs of modern students. To improve students' learning efficiency and strengthen the teaching effect, many teachers have introduced English language learning model matching in their teaching activities, the central idea of which is to develop different teaching programs for different students in order to promote the progress of students with different learning abilities. English language learning pattern matching is a common teaching model in which students are divided into different groups of similar levels according to their knowledge and learning ability, and teachers make different teaching plans according to the differences between the groups. It is a common teaching model used in many universities and has been very effective and popular among educators [15, 16].

For the English language learning pattern matching problem, the clustering algorithm is a simple, efficient, easy-to-implement, and scalable clustering analysis method, which is widely used in machine learning, image analysis, pattern recognition, data compression, and other fields. The clustering algorithm is of great significance for English language learning pattern matching. However, with the advent of big data era, the scale of data grows rapidly, and traditional algorithms face the challenges of large sample data and high feature dimensionality. Therefore, it is of great importance to design algorithms suitable for a big data environment. With the widespread use of distributed frameworks for algorithms, the MapReduce big data computing model developed by the Apache Foundation is favored by researchers for its high stability and scalability. Among them, the parallel K-means algorithm is based on MapReduce, but the algorithm does not consider the problem that high-dimensional data has many unrelated features while the structure is sparse, and direct clustering will lead to poor clustering results.

To solve this problem, the *K*-means algorithm based on MapReduce and locally sensitive hashing LSH maps the high-dimensional data into data buckets and compresses the similar data in the data buckets into databases, thereby reducing the redundant computation of similar data and improving the clustering effect. However, the algorithm does not directly process the data with features and only weakens the influence of high-dimensional data on the clustering effect by fusing similar data, and the clustering effect is improved to a limited extent. Based on MapReduce platform and nonnegative matrix decomposition NMF, the MR-KNMF algorithm is proposed to decompose the high-dimensional data matrix into a nonnegative basis matrix and coefficient matrix, and finally replace the original high-dimensional matrix with a low-dimensional coefficient matrix for clustering. The algorithm effectively reduces the dimensionality of the data, but the effect of irrelevant features on the clustering effect is not considered before the nonnegative matrix decomposition, which leads to the reduced clustering effect of the algorithm.

The main contributions of this study are as follows. In recent years, with the continuous reform of the new curriculum standards, the education system and teaching philosophy have been continuously improved and perfected. Under the background of quality education, the traditional teaching methods can no longer meet the learning needs of students, and today's education and teaching are not only the transmission of theoretical knowledge, for English, teachers should fully clarify their teaching objectives as well as teaching tasks, so that students' English ability in the learning can be improved. The core literacy of English has four main aspects: language ability, cultural awareness, thinking quality, and learning ability, so the teacher should closely focus on these four aspects in the actual teaching to cultivate students' core literacy, so as to effectively match the English language learning mode, complete the expected teaching effect, and reflect the construction of an efficient classroom. This study proposes a MapReduce-based parallel clustering algorithm for English language learning pattern matching based on the application of the clustering algorithm in the framework of big data and addresses the problems of poor clustering effect in the face of high-dimensional data, uneven data partitioning, and sensitivity of initial prime in the parallel clustering algorithm in the big data environment. The relevant experiments show that the method can be accurately applied to actual English teaching.

## 2. Related Work

### 2.1. English Language Learning Model Matching

English language learning pattern matching is scientific, planned, relevant, and flexible in practice, and its rational application can not only improve students' learning ability, but also promote students' individual development, create a good learning atmosphere for students, and allow students of different levels to adjust to the best learning state. This is an important trend in the future of teaching and learning.

English language learning pattern matching is not just a simple matching of teaching contents, but also a hierarchy of actual teaching goals. Teachers can also design and teach the English language with the core purpose of achieving the teaching objectives. In this regard, teachers can also set different levels of goals for students' basic differences and adopt a progressive strategy to help students gradually improve their English proficiency and application skills through a combination of stage goals, semester goals, and annual goals. In meeting students' individual learning needs, we strengthen students' training in special exercises to improve their English level in a planned way [17–19].

Nowadays, English teaching not only needs to improve students' basic English level, but also needs to improve students' English application ability and, at the same time, it needs to play the role of English teaching to establish morals and educate people. In the process of designing teaching objectives, teachers need to design teaching tasks according to the teaching content and the needs of students' vocational job abilities, and guide students to complete teaching tasks independently, analyze, summarize, and conclude knowledge, enhance students' independent learning ability, and play a role in promoting students' future development. Teachers need to pay attention to teaching design in the process of teaching, scientific, and reasonable teaching design to improve the quality of classroom teaching and stimulate students' learning potential which plays an important role. The English course is not only to improve students' English knowledge but also to combine students' professional development to provide service capacity for students' development. The implementation of curriculum thinking and government in English teaching improves the role of English teaching moral education, to improve students' comprehensive literacy; professional competence and teaching need combined humanistic literacy and moral character to the combination.

Teachers teaching process using English language learning mode matching, according to the characteristics of students' set teaching objectives, enhances the students' sense of joy in learning English, which plays a guaranteed role in enhancing students' interest in learning English, in the process of learning, so that students are clear about the practical problems that the English knowledge learned can solve, enhance the application of students' knowledge, so that students can complete teaching tasks independently, enhance the student's sense of joy in learning, and effectively enhance students' interest in learning. The goal of English teaching reform is to improve students' interest in learning English, focus on the cultivation of students' vocational ability, comprehensively improve students' ability to apply English knowledge, and play the role of English teaching in students' growth process. Therefore, teachers need to pay attention to the cultivation of students' interest in English language learning mode matching, design interesting forms of learning activities, choose students' favorite learning contents, and seize students' curiosity and curiosity, so that they can develop an interest in the actual learning of English language learning mode matching and be willing to devote themselves to learning.

In addition, under the Internet-based English language learning mode matching, students have more time for online self-learning and self-learning has become an indispensable part of English teaching, so teachers need to focus on teaching learning skills in the classroom, regulate students' learning behaviors, guide students to explore learning methods with their own characteristics, conduct teaching exploration according to students' characteristics, and cultivate students' good habits. The teachers need to focus on teaching learning skills in the classroom, regulate students' learning behaviors, and guide students to explore learning methods according to their own characteristics. In the process of cultivating students' abilities, we need to pay attention to the improvement of students' quality. The integration of curriculum thinking and politics into English teaching highlights the role of moral education and plays a basic role in guaranteeing the improvement of students' comprehensive ability. In short, according to the talent training objectives, the traditional English teaching mode is not suitable for the current needs of student development, and practice has proved that college English adopts English language learning mode matching with the current teaching reform needs, and also with the needs of students' English level. In the process of teaching reform, we should focus on the needs of students' vocational positions, center on students' development, improve students' application ability of basic English knowledge, promote students' professional development, and train students into senior technical talents who meet the needs of modern enterprises [20–22].

### 2.2. Big Data Clustering Technology

At present, with the development of the Internet, the accumulation of data volume has been accelerated, and the text data among them have high dimensionality and large data volume, and at the same time records valuable information; therefore, text clustering methods can be used to categorize and process similar texts, and extracting key information in the text can provide the basis for intelligent recommendation and many other fields, so as to realize the effective use of resources. Traditional serial text clustering methods are difficult to process efficiently for massive text data. With the emergence of cloud computing technology, the implementation of distributed text clustering under the Hadoop platform is a direction worth studying.

The *K*-means clustering algorithm is implemented in parallel under the Hadoop platform, and the parallel clustering is implemented in an iterative manner for massive data by MapReduce. The parallelization improves the *K*-means method and experiments show that the method can reduce overhead while increasing efficiency. Using the maximum minimum distance to determine the initial center of the clustering method, the shortcomings of *K*-means, which are sensitive to the initial value, can be eliminated, and it can be implemented under the Hadoop platform, which can consume less time. The Hadoop platform is an open-source framework for implementing distributed computing, which consists of two main parts, HDFS, and MapReduce programming framework.

The Name Node in HDFS is mainly responsible for the namespace of the distributed file system. The Map function cuts the input data into small independent data blocks in the form of key, value and outputs them in the form of key and value, and provides them to the Reduce function at the same time; the Reduce function receives the intermediate results of Map processing, merges the same keys, and values, and finally outputs the results and writes them to HDFS [23–27].

Although the poor clustering effect in the face of high-dimensional data seriously affects the algorithmic performance of parallel *K*-means, the impact of data partitioning on parallel *K*-means cannot be ignored. At present, the MapReduce-based *K*-means algorithm usually uses the default data partitioner for data partitioning, which is prone to data skewing, making the load of each node unbalanced and reducing the algorithm running efficiency. Therefore, to divide the data evenly, the parallel division clustering algorithm MR-PGDLSH based on the MapReduce framework determines the dispersion of the data by calculating the error squared and SSE through an adaptive grouping strategy and finishes dividing the data evenly when the SSEmin is reached. SBASC, a sorting and balancing algorithm for scalable simple random sampling, first samples the data and then sorts the data based on the sampling results, thereby dividing the neighboring data into the same partition and thus achieving data balancing. Although these algorithms have improved data partitioning, they still suffer from high time complexity and uneven data partitioning. In addition, the MapReduce-based *K*-means algorithm suffers from the initial center-of-mass sensitivity problem, and the randomly selected center-of-mass tends to make the algorithm fall into local optimum. By introducing Canopy to coarsely cluster the dataset in advance, multiple overlapping Canopies are generated to obtain a more accurate center of masses, so that the algorithm terminates in a global optimal solution [28–30].

However, the use of manually determining the radius of the Canopy can make the clustering effect unstable. By merging neighboring cluster cores and neighboring clusters and calculating the newly merged cluster cores as the center of mass for that iteration, the manual determination of radius is avoided, but the problem of algorithm instability is not completely solved. Meanwhile, the swarm intelligence algorithm has been widely used to obtain the optimal solution to the problem by simulating the self-organization behavior of organisms, and many scholars began to combine the swarm intelligence algorithm to improve the problem of center-of-mass sensitivity. Using adaptive cuckoo search ACS to optimize *K*-means, the MR-ACSKMC algorithm is proposed to improve the search ability of cuckoo populations by adaptive step size to obtain the global best center of mass. The algorithm MRGAPKCA, which combines genetic algorithm and *K*-means, is proposed in the Hadoop environment to optimize the selection of center of mass and improve the global search ability of the algorithm. The above algorithm successfully combines the swarm intelligence algorithm with a parallel *K*-means algorithm to improve the problem of initial prime sensitivity, but there are still disadvantages of slow local convergence and difficulty in obtaining the global optimum, and the problem of initial prime sensitivity needs further improvement.

## 3. Methods

### 3.1. Model Architecture

**Figure 2:**
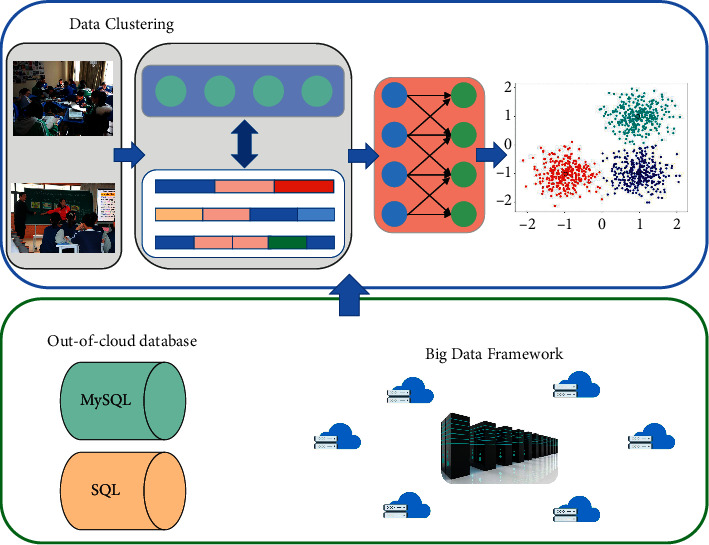
Model structure.

The Hadoop platform is an open-source project developed by Apache Software Foundation. The core components of the platform are MapReduce and HDFS. HDFS stores the divided data blocks in a distributed form into multiple nodes. The work of the name node is used to manage the namespace and handle operations related to client access to data; the work of the data node is used to complete the storage of the node, according to the requirements of the name node to achieve the creation and deletion of data blocks and other operations. MapReduce is the core of the map and reduces functions, through them to achieve the parallelization of large amounts of data programming. In MapReduce, a new key-value pair is obtained as the intermediate output after the mapping of the map, and the intermediate result is processed by the reduce function to obtain the result. The structure of the model is shown in Figure 2.

### 3.2. Feature Embedding

First, the input sentences are initially encoded into a 128 × 384 word vector matrix by embedding layer, where 128 is the fixed length of the sentence and 384 is the dimension of the word vector, and the text information of the input model needs to include the location information of the words, because the meaning of the sentences composed of the same words may be completely different. In order to cope with this situation, the BERT model adopts the position-coding method shown in the following equation:

(1) $$\begin{array}{c} PE\left(pos,2i\right)=\sin\;\left({\frac{pos}{10000}}^{2i/d_{mode\;l\;}}\right),\\ PE\left(pos,2i+1\right)=\cos\;\left({\frac{pos}{10000}}^{2i/d_{mode\;l\;}}\right), \end{array}$$

where pos is the corresponding position of the word in the sentence; *i* is the position parameter; odd position is calculated using the equation, even position is calculated using the equation, model is the position matrix dimension, and the position information of the word can be obtained by summing the sine and cosine waves of the period change. In this study, to simplify the computational process, we choose to set the position encoding layer directly as a trainable matrix and let it join the iterative operation of the whole neural network, after which the position matrix is summed with the word embedding matrix and sent to the hybrid attention network as input information for training. The text features are first extracted using the hybrid attention module, then the features are learned in the residual connected normalization layer and the convolutional connected layer to obtain more text information, and then they are fed to the next hybrid attention module for further learning. In this study, a 12-layer hybrid attention network is used as the main body of the model, which minimizes the parameters without excessive loss of accuracy due to the use of layer parameter sharing. Finally, SoftMax is used in the output layer to obtain the probability information of text classification and to accomplish the text classification goal.

### 3.3. Word Vector Convolution Module

To improve the effect of processing Chinese text and obtain better word vector features, this study combines the attention mechanism and proposes the uniform word vector convolution module AWC: first, four matrices are combined into one matrix *T*, and then the matrices *V*_1_ and *K*_1_ are obtained by two linear layer transformations, respectively, and its calculation formula is shown:

(2) $$\begin{array}{c} V_{1}=TW_{av},\\ K_{1}=TW_{ak}\text{,} \end{array}$$

where **W**_*av*_ and **W**_*ak*_ are the connection matrix; **T** matrix is linearly transformed to get *V*_1_ and *K*_1_ matrices, respectively; *V*_1_ represents the word vector matrix; and the *K*_1_ matrix represents the query matrix, after which the attention score is calculated for *V*_1_ and *K*_1_ matrices, and the formula is shown in the following equation:

(3) $$\begin{array}{c} Atten\left(V_{1},K_{1}\right)=softmax\;\left(f\left(V_{1},K_{1}\right)\right)V_{1}. \end{array}$$

The *f* in the equation is a function by which the correlation between *V*_1_ and *K*_1_ is calculated to obtain the most important part of the four-word vectors, and the equation is the *f* function calculation formula:

(4) $$\begin{array}{c} f\left(V_{1},K_{1}\right)=\tanh\;\left(V_{1}K_{1}\right)W_{at}, \end{array}$$

where **W**_*at*_ is a learning matrix, which can assist **V**_1_ and **K**_1_ matrices by subsequent learning to obtain more accurate word vector information. After the attention operation of the above equations, it is possible to obtain rich word vector features, which have better results than the algorithm.

### 3.4. MSSA Algorithm

After uniform partitioning of the data, the partitioned data blocks are input into the MapReduce task for parallel clustering. The current MapReduce-based K-means algorithm needs to initialize the prime when performing parallel clustering, and an improper initial prime can cause the algorithm to fall into a local optimum, so the algorithm is sensitive to the selection of the initial prime. Since the SSA algorithm has good global search ability and excellent search ability, the introduction of the SSA algorithm is considered to improve the sensitivity of the parallel K-means algorithm to the initial center of mass, but the algorithm will gradually decrease in diversity during the iteration and may converge too early. To this end, this study proposes the nonuniform variational sparrow search algorithm MSSA and then uses MSSA to optimize the prime finding process and obtain the final clustering results. The main steps of MSSA algorithm implementation are: (a) Leading sparrow search: propose nonuniform leading sparrow*X*_*ij*_′, and use nonuniform operators to perturb the position of the leading sparrow to increase the population diversity. (b) Follower sparrow following. The adaptive coefficients are used to improve the following step of the follower sparrow, and the adaptive follower sparrow *X*_*ij*_ is proposed to update the follower sparrow position.

In the SSA algorithm, the leading sparrow leads the whole population movement and will lead the sparrow population to search again after reaching the warning value, which can jump out the local optimum well. However, before the warning value is reached, the diversity will gradually decrease with the iteration of the algorithm. To make the algorithm always maintain the global search ability, nonuniform variation leading sparrow *X* is proposed, and the *j*th dimensional component of the generated leading sparrow *X* is nonuniformly varied. The nonuniform variation leading sparrow is *X*_*ij*_′. It is known that Δ(*t*, *UB* − *X*_*ij*_) is the nonuniform variation operator, *UB*, *LB* are the upper and lower bounds of *X*, respectively, *t* is the number of iterations, and the nonuniform variation leading sparrow *X*_*ij*_′ is defined as follows:

(5) $$\begin{array}{c} X_{ij}^{\prime }=\left\{\begin{array}{cc} X_{ij}+\Delta \left(t,UB-X_{ij}\right)\text{,} & if\;a\;random\;\xi \;is\;0\text{,}\\ X_{ij}-\Delta \left(t,X_{ij}-LB\right)\text{,} & if\;a\;random\;\xi \;is\;1\text{.} \end{array}\right. \end{array}$$

In the SSA algorithm, the follower sparrow jumps directly to the vicinity of the current optimal solution, and when the leading sparrow is poorly positioned, the algorithm converges quickly to the local optimum, which is not conducive to the algorithm obtaining the global optimum. Therefore, the process of following sparrow following is improved using adaptive coefficients, and adaptive following sparrow *X*_*ij*_^*t*+1^ is proposed. The best position *X*_*p*_^*t*^ of the *t*th iteration is known, *A* is the adaptive coefficient, *α*_*t*_ is a variable decreasing linearly from 2 to 0 about the number of iterations *t*, Υ is a vector consisting of [0, 1] random numbers, and *C* is a coefficient vector, then an adaptive following sparrow *X*_*ij*_^*t*+1^ is defined as follows:

(6) $$\begin{array}{c} X_{ij}^{t+1}=\left\{\begin{array}{c} Q\cdot \exp\;\left(\frac{X_{worst}^{t}-X_{ij}^{t}}{i^{2}}\right),if\;i>\frac{n}{2},\\ X_{p}^{t}-\left(2\alpha_{t}\Upsilon -\alpha_{t}\right)\cdot \left|CX_{p}^{t}-X_{ij}^{t}\right|,otherwise\text{.} \end{array}\right. \end{array}$$

The MSSA algorithm process is as follows: (a) sparrow population initialization; (b) calculate the fitness of the sparrow population and select the best position and the worst position; (c) select the leading sparrow and the follower sparrow according to the fitness value. The leading sparrow position is updated using the equation and then perturbed using the equation. Subsequently, the positions of the follower sparrow and warning sparrow are updated using the equation, respectively; (d) recalculate the sparrow fitness; and (e) determine whether the end condition is reached and turn to (b) if it is not, and the algorithm ends if it is reached.

### 3.5. Parallel Clustering

**Figure 3:**
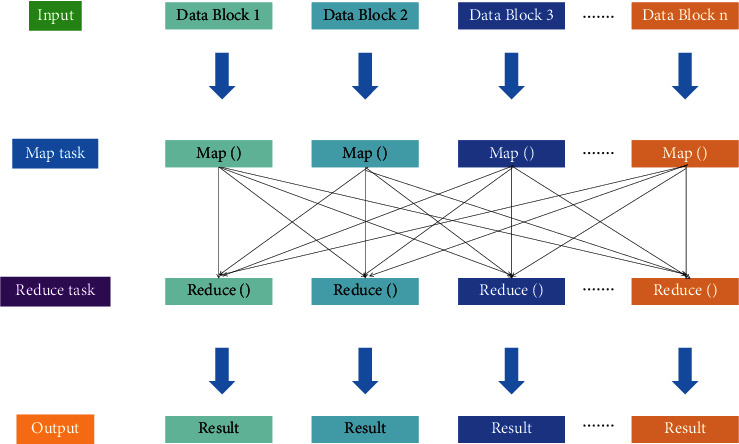
Principle of parallel computing in MapReduce model.

After proposing MSSA, the performance of MSSA with good global search ability and strong merit-seeking ability can be used in combination with parallel K-means to process the data together to finally achieve parallel clustering. The specific steps are as follows: (a) the dataset is viewed as a group of sparrows *S* and the basic parameters are initialized: population size N, number of leading sparrows *PD*, number of warning sparrows *SD*, warning value *R*_2_, and maximum number of iterations *T*. (b) Parallel partitioning clustering is performed in MapReduce framework. In the Map stage, the Euclidean distance from other sparrows to the leading sparrow is calculated in parallel as Value, and the composed key-value pairs are passed to the Reduce task. (c) In the MapReduce framework, the fitness of each sparrow is calculated in parallel. The adaptation size is taken as the reciprocal of the distance from the sparrow to the center of mass. The adaptability is calculated by taking the leading sparrow's subscript number as Key and the adaptability size as Value, and then sorted according to the adaptability. (d) The top *PD* sparrow with the best adaptability is selected as the leading sparrow, and the rest are the follower sparrows. After updating the position of the leader sparrow in parallel using the equation, the position of the follower sparrow is updated in parallel using the equation, and then the position of the follower sparrow is updated in parallel according to the equation. (e) The *SD* sparrow is randomly selected as the warning sparrow, and the population is warned with the warning value *R*_*2*_ as the judgment condition, and the position is updated in parallel according. (f) After one iteration is completed, the maximum number of iterations is judged. If the maximum number of iterations is not reached, we move to step (b), and when the maximum number of iterations is reached, the algorithm ends, and the final clustering result is output. The principle of parallel computing in the MapReduce model is shown in Figure 3.

## 4. Experiments and Results

### 4.1. Experiment Setup

The experimental environment in this study is set up as a Hadoop computing cluster with one Master node and three Slaver nodes; all hosts have 8-core CPUs, 16 GB RAM, and 512G hard disk. Each host was installed with Hadoop version 2.7.7, operating system CentOS 6.0, JDK version JDK1.8.0, and 1 GB/s Ethernet connection between hosts.  Table 1 describes the configuration of each node.

**Figure 4:**
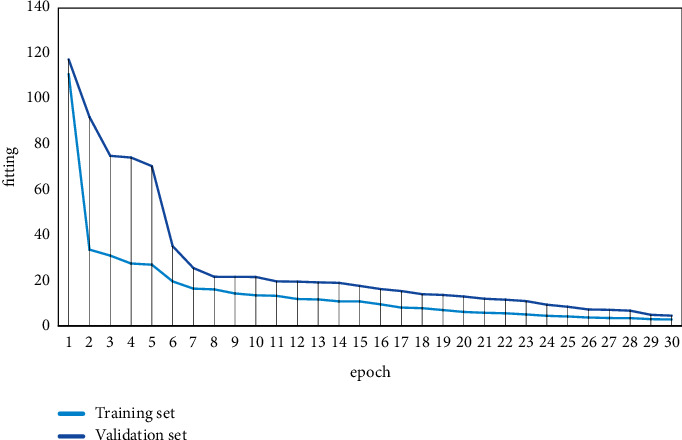
The training process loss convergence schematic.

**Figure 5:**
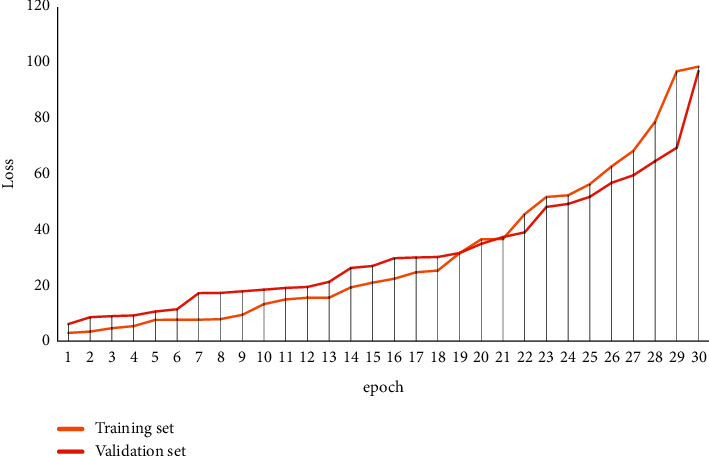
Schematic diagram of training process performance improvement.

The experimental datasets were selected from the English language learning pattern matching teaching collected from several Chinese universities and constructed manually. The size of the three constructed datasets, Dataset 1, Dataset 2, and Dataset 3, was 100, 1000, and 5000, respectively, and the corresponding classification numbers of the three datasets were 3, 5, and 10. The parameters are Gmax = 300, *P* = 0.92, *P*_*m*_ = 0.02, and the values of *P*_*c*min_, P_mmin_, *P*_*c*max_, and *P*_*m*max_ in the adaptive operator are 0.1, 0.05, 0.9, and 0.1, respectively. The experiments were tested in terms of clustering accuracy and clustering efficiency, respectively. The training process loss convergence and performance enhancement are shown in Figures 4 and 5.

### 4.2. Experimental Results

**Figure 6:**
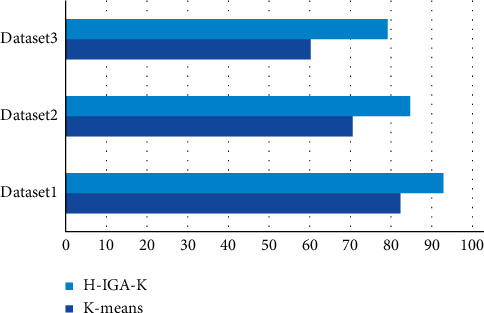
Comparison of clustering accuracy of different datasets.

To test the clustering accuracy of the Hadoop-based improved MSSA clustering algorithm, the data in the experimental dataset were processed using the Hadoop-based K-means algorithm and the H-IGA-K algorithm, and the clustering accuracies of both are given in Figure 6. As shown in Figure 6, the clustering accuracy of both algorithms decreases as the data size increases, but the degree of decrease is different, and the H-IGA-K algorithm has higher clustering accuracy than the traditional Hadoop-based K-means clustering algorithm in any dataset, especially the Dataset 3 dataset, thus indicating that the improved Hadoop-based MSSA clustering algorithm outperforms Hadoop-based *K*-means clustering algorithm and is suitable for handling large-scale datasets.

**Figure 7:**
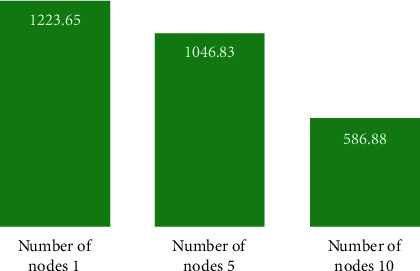
Dataset 1 test dataset runtime for text clustering at different nodes.

**Figure 8:**
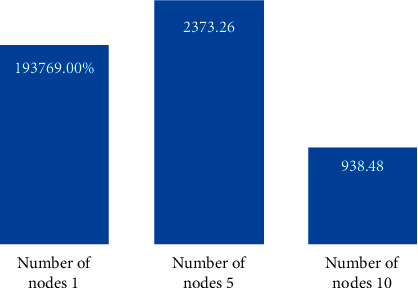
Dataset 2 test dataset runtime for text clustering at different nodes.

**Figure 9:**
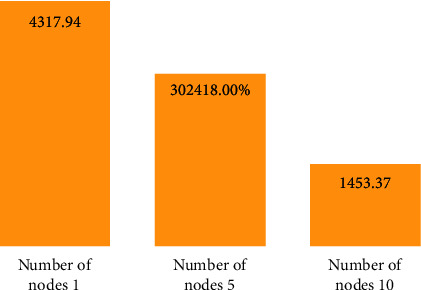
Dataset 3 tests the runtime of the dataset for text clustering at different nodes.

In order to test the clustering efficiency of the improved Hadoop-based MSSA clustering algorithm, this experiment was conducted on three test datasets, Dataset 1, Dataset 2, and Dataset 3, on clusters built with 1, 5, and 10 nodes, respectively, and Figures 7–9 give the results of the improved Hadoop-based MSSA clustering algorithm. Dataset 2 and Dataset 3 on a different number of nodes for text clustering in comparison to the time required to run.

As seen in Figures 7–9, the total time required to run the program decreases the more the number of nodes in the Hadoop cluster for Dataset 1, Dataset 2, and Dataset 3, the more the number of nodes increases, the less the time required to run the program, and the more the size of the dataset increases, the more the time consumed to run the algorithm decreases, thus indicating that the Hadoop-based cluster with multiple nodes is more suitable for processing large datasets, especially when the number of nodes reaches 10, the algorithm runs much more efficiently.

The experimental platform was built using eight identical computers. The 48-dimensional datasets with sizes of 16G and 32G were constructed. In the constructed datasets, the attributes of the data have a greater impact on the classification. The Hadoop-based K-means algorithm and the Hadoop-based MSSA algorithm were tested in terms of convergence speed and clustering accuracy, and the comparison results of convergence speed and clustering accuracy are given in Tables 2 and 3, respectively.

As seen in Tables 2 and 3, the average number of iterations of the Hadoop-based MSSA algorithm is the smallest and the accuracy of the clustering results is higher when operating on 16G and 32G datasets, respectively, indicating that the Hadoop-based MSSA algorithm is better than the Hadoop-based K-means algorithm and more suitable for processing data with large datasets, which is due to the fact that in the H-MSSA algorithm, the influence of attribute weights is considered.

In this study, we propose an improved genetic clustering algorithm and implement the improved genetic clustering algorithm based on the Hadoop platform to address the problem of low efficiency of clustering massive data by single machine serial. Experiments show that the improved algorithm outperforms the classical clustering algorithm and has a large improvement in clustering accuracy and clustering efficiency, which is suitable for processing large-scale datasets.

## 5. Conclusion

In the English classroom, to eliminate the differences among students, it is necessary for teachers to match the English language learning patterns, so as to achieve the individual development of each student, truly reflect the diversity of classroom forms, complete the effective penetration of core literacy, and thus improve the overall quality of students. In the English classroom, teachers should be aware of the importance of matching English language learning modes, and can continuously penetrate core literacy education to achieve the expected teaching effect and improve students' comprehensive quality; in this process, teachers need to change the teaching status quo, give full play to the main role of students, and combine a variety of teaching methods to reflect diverse classroom modes, so as to complete the teaching of quality education. In this process, teachers need to change the teaching status quo, give full play to the students' main role, and combine a variety of teaching methods to reflect a variety of classroom models, so as to achieve the goal of quality education, so that students can develop in all aspects.

By analyzing the big data feature extraction method based on the large-scale English language learning pattern matching data and the Hadoop platform as the technology, we not only can effectively complete the clustering processing analysis of English language learning pattern matching data and provide the basis for user feature data extraction, but also design the data processing method with good scalability, which can effectively analyze and extract the user feature data. The designed data processing method not only can effectively complete the analysis of English language learning pattern matching data and provide a basis for user feature data extraction, but also has good scalability to effectively analyze and extract user feature data, so as to obtain key feature data of users in the big data environment. In the future, we plan to develop a big data framework using recurrent neural networks with clustering algorithms in English language learning pattern matching.

## Figures and Tables

**Table 1:** Experimental environment.

Node type	Host name	IP address
Master	Master	192.168.111.1
Slave	Slave 1	192.168.111.2
Slave	slave_2	192.168.111.3
Slave	Slave 3	192.168.111.4

**Table 2:** Comparison of convergence speed.

Dataset (G)	Algorithm name	Average number of iterations
16	*K*-means algorithm	11
	H-IKA algorithm	6

32	*K*-means algorithm	16
	H-IKA algorithm	7

**Table 3:** Comparison of clustering accuracy.

Dataset (G)	Algorithm name	Clustering accuracy (%)
16	*K*-means algorithm	87.47
	H-IKA algorithm	93.85

32	*K*-means algorithm	72.28
	H-IKA algorithm	92.19

## Data Availability

The data used to support the findings of the study are available from the corresponding author upon request.
